# A Glance at the Hospitals of Berlin and Vienna

**Published:** 1858-12

**Authors:** 


					﻿A Glance at the Hospitals of Berlin and Vienna—Treatment of Frac-
tures by Envelopment in Plaster of Paris—General Remarks. By
Geo. Suckley, M. D.
During a hasty visit at the capitals of Prussia and Austria, a few months
ago, I observed some facts in connection with their hospitals, which may
perhaps be of interest to the readers of your Journal.
These observations, although made while I was rapidly moving from place
to place, seem of sufficient interest to excuse me for attempting to com-
municate what could be so much more fully given by those whose longer
sojourn at the cities named, render them much better prepared than my-
self, but who, from one cause or another, “ hide their lights under a bushel,”
and remain silent.
The Charity Hospital of Berlin is a very large, well-organized institution,
under the direct patronage and care of the Government. Its Winter wards
are generally about half the size of the principal surgical wards in the main
building of the N. Y. Hospital, but those for Summer (in a fine building
specially reserved for use during the warm season) are quite large and airy.
In the Winter buildings there is an attempt at artificial ventilation of the
apartments, which, although of evident benefit, is scarcely equal to the sys-
tem at the Beaujon Hospital, in Paris, and in my opinion far inferior to the
thorough methods in use in the first class hospitals and other public institu-
tions of our own country. The hospital also, as a whole, seems to me to be
far superior to the average of those under the Paris administration. Like
those of the latter, everything is systematized, but there appears to be more
of comfort in the arrangements—-a something in the general order of things,
which seems to convey the impression that the institution is for the taking
care of sick human beings, rather than of sick machines.
At meal time, a portion of every article cooked is placed upon a table, in
the public kitchen, upon which also stands a sample of each kind of wine, or
extra article of luxury, intended for the sick. It is the duty of an inspector
to taste some of each before the provisions are distributed to the different
wards. When visitors are present, they are politely asked by the inspector
to partake of some of the articles; which request, in my own case, was com-
plied with. I found all the meats, &c., as well cooked and seasoned as if
for a private family, and the wines, beer and spirits of a fair quality.
The wards for the sick contain usually 13 beds each, which are not cov-
ered up by the close, unhealthy, heavy curtains, so constant in the Parisian
hospitals, but open, as in our own.
Although I visited the whole of the institution, I spent the most time in
the surgical wards, especially in those attached to the service of that estima-
ble old surgeon, Fungken. The practice here I found very much resembles
that in the first class American institutions. The surgeons of Berlin are not
weighed down by the incubus of conceit and egotism, which so hampers the
practice of the Parisians. They seem to draw good from all sources, and to
be readers and students of the practice of all countries, keeping up with the
progress of the age; trying all new discoveries which seem reasonable, and
rejecting those found worthless. In Fungken’s service I saw Chassaignac’s
“ drainage tubes” in common use. The employment of small bathing tubs
for soaking inflamed arms and legs, and the use of oiled silk for protecting
beds, covering dresses, splints, &c., put me much in mind of our own
practice.
I had previously heard of the Berlin method of treating fractures bg
encasing the limb in Plaster of Paris, and was particularly fortunate in be-
ing present at the time a recent fracture of the leg was thus treated. The
method is said so have been introduced at Berlin about 40 years ago, having
been brought from the Cossacks, Caucasians, or Tartars, (I have forgotten
which.) It gives great satisfaction to the Prussian surgeons, and seems
well adapted to the treatment of fractures, under peculiar circumstances,
such as when the patient resides at a long distance from the surgeon, or
when, from restlessness, delirium tremens, or some other cause, bandages
and ordinary splints cannot be kept properly adjusted. The process, as I
saw it applied, was as follows: The limb was first bandaged with many-tail-
ed bandages of flannel, which had been previously spread with simple
cerate or lard; then enveloped with another bandage, covered with dry pow-
dered gypsum, and neatly applied. The patient, a hearty laboring man,
having been thoroughly relaxed by chloroform, was then subjected to strong
manual extension and counter-extension, until the fragments of the injured
lmb were brought in their proper relations. The limb was then placed in
a trough resembling a fracture box, the sides of which, being on hinges,
could be let down at will; and the extension having been maintained by an
assistant, a sufficient quantity of plaster of paris, mixed cold as if for taking
tcasts, and of about the consistence of thin “ mush,” was then poured in the
box, enveloping the leg, ancle, and instep. In a few minutes this became
sufficiently hard to allow the extension to be dispensed with, and in a short
time longer the whole bad consolidated so much that the sides of the mould-
ing box were let down, and the casing left to dry rapidly in the atmosphere.
The thickness of the casing averaged about three-fourths of an inch, and
seemed, when dry, sufficiently strong to resist any violence to which a pa-
tient is ordinarily subjected, while awaiting the consolidation of a fracture.*
Before the casing becomes perfectly dry, the date of the “ putting up ”
of the fracture is scratched upon it. It is left on for four or five weeks, and
when removed is broken with a hammer and chisel. Should small ulcers
occur, or any inflammation about the point of injury, threatening the con-
version of a simple into a compound fracture, a small circular hole is “ chip-
ped ” out with a chisel, in order that the part can be examined, and, if
necessary, the proper dressings applied.
This method of treating fracturesis applied only to those of the leg and
forearm. It is thought to be highly preferable to the starched bandage, in
such common use in other cities, as the plaster in drying does not shrink.
The case in which I witnessed the application of this treatment was that of a
simple fractuie of both bones of the leg, of only 16 hours standing.
The Prussian surgeons, like those of Paris, as an anaesthetic, use chloro-
form altogether, having abandoned sulphuric ether as tedious and disagree-
able, and as not possessing sufficient advantages in any way over the first to
warrant its substitution.
In the Charity Hospital, the general arrangements of the wards, the dres-
sings, baths, cleanliness, and conveniences generally, reminded me much of
the state of affairs at present in vogue in our New York hospitals, and seem
far superior to those of the French.
There are many advantages for students at Berlin; and as Americans and
other foreigners are not, as in Paris, a drug in the market, they receive
much attention, and have as good, and in my opinion better chance to bene-
fit themselves by studying European practice, than in any other of the great
continental citie>; apparently much better than is to be had at Vienna, to
which place Americans, especially Bostonians, are beginning to give their
attention.
The great public hospital at Vienna is a very large institution, containing
wo thousand or more beds. As a school for diseases of the eye and skin,
*The limb should not be allow to rest at the bottom of the box or trough at the
time the plaster is poured in, but should be kept up a short distance, to allow an
amount to find its way behind the limb, sufficient to render the casing over the calf
of the leg and posterior portion of the limb generally, as thick and strong as that
which covers the front. To facilitate the equal spreading of the mixture, the box
hould be tapped with a small wooden hammer while the plaster is still fluid.
it is celebrated; and as a hospital for the study of diseases generally, it has a
great reputation on the Continent. It is supported by the Austrian Gov-
ernment, and on the whole appears to be well managed; although, owing to
the financial embarrassment of Government, it is not nearly so well conduc-
ted or so liberally supported as it would otherwise be. It is not so neat as
the Berlin Hospital, and the bed-clothes and patients had, to my eyes, a
dirty appearance. Many facilities are furnished medical students, and in-
struction in all branches is abundant and cheap. The steam baths and the
arrangements for bathing, &c., on a grand scale, are well worthy of atten-
tion. As at Berlin, the cooked food and other edibles are daily inspected
before distributing to the sick; each physician or surgeon taking turn in act-
ing as inspector, instead of the business being attended to by a permanent
inspector, as at Berlin. Living is cheap at either place, and the facilities fur-
nished foreign students apparently as great as at Paris.—American Medi-
cal Gazette.
New Operation for Ranula.—The following operation, a notice of which
we find in the Gazette des Hbpitaux, tor the cure of ranula, proposed by
M. Barrier, of Lyons, recommen Is itself for its ingenuity and simplicity,
and is worthy of a trial in a disease which often baffles the skill of the sur-
geon. Each extremity of the transverse diameter of the tumor is seized by
a pair of forceps held by assistant. The operator taking that on the left
side himself, cuts with the scissors a triangular flap, whose base is to the
right of the antero posterior diameter, the apex (which should be truncated)
being consequently to the left. He then takes the other forceps, and makes
a small incision from Lefore backward, near the base of the flap, extending
through the cyst. Through this slit is inserted the apex of the flap, which
is turned inward, from left to right, and is secured by a suture. In this
way the mucous membrane, being turned inward, forms the lining of the
sac, which consequently does not tend to close, but allows the free escape
of its contents.—Boston Medical and Surgical Journal.
A Singular Muscle. Observed by Marion Howard, M. D., Demonstrator
of Anatomy, Medical College of Virginia.
Some time last winter, one of the students, while dissecting, asked me
“ Doctor, what muscle is this? I don’t understand it.” On examination it
turned out to be a very eccentric muscle indeed; arising by two fleshy
bands about an inch broad from the posterior edges of the tibia and fibula,
about an inch above the lower extremities of the bones, joining on the
median line, and enclosing in the bands the tendons of all the muscles of
the posterior part of the leg except the tendon-achillis, and sending otf from
the point of junction a round fleshy belly about half the size of the little
finger, which passing behind the inner ancle and under the inner edge of
the os calcis, from the larger tuberosity of which, it received a fleshy fascicu-
lus, became tendonous as it turned forward in the sole of the foot, and joined
the massa carnea at its anterior edge. In the other leg there was no such
muscle.— The Virginia Medical Journal.
A Case of Obstruction of the Bowels relieved by Copious Injections, after
the Failure of other means. By 0. C. Gibbs, M. D.
The following case is interesting, because relief came when but little expec-
ted, and under desperate circumstances:
January lsZ, 1858.—I was called to see Mrs R-------------, aged about 55
years. She complained of severe pain in the bowels, was vomiting some,
the pulse was quiet, tongue furred, bowels tender on pressure, the counten-
ance haggard and indicative of much distress and prostration. I suspected a
a strangulated hernia, but, on inquiry and examination, this supposition
proved groundless. Opium, in full doses, combined with small doses of
calomel, was advised internally, and hot fomentations locally.
January 2.d.—The patient was suffering less pain, but not otherwise im-
proved. The treatment was continued, the opium in diminished, and the
calomel in increased, doses.
January 3d.—The patient was no better; the pulse was more frequen
and the vomiting still continued. Castor oil was now ordered in tablespoon-
ful doses, to be repeated every hour until it operated; the action of the oil
to be aided by injections of infusion of senna.
January 4th.—Still no improvement, but patient gradually failing;
Bowels were much bloated and tympanitic. The matters vomited now were
stercoraceous. Cloths, saturated in turpentine, were applied to the bowels,
calomel, rubbed up with blue-pill, was now ordered, in four grain doses of
the mass, to be repeated every two hours.
January 5th.—Treatment had been discontinued during the night; the
friend supposed the patient dying, and refused to give additional medicines.
The patient vas evidently sinking; the bowels were greatly distended, the
vomiting stercoraceous, no operation upon the bowels had yet been secured.
Though the case was unpromising, I urged the friends to additional efforts.
A blister was ordered over the stoYnach; as soon as drawn, the cuticle was
directed to be removed, and one grain of morphine to be applied to the
denuded surface; turpentine was still ordered to the bowels; and calomel,
in five grain doses, to be administered every three hours.
January 6th.—Still no operation from the bowels, and no cessation of
the vomiting. The patient was slowly sinking. Ordered treatment con-
tinued, though, I must confess, with no hopes of relief.
January 7th.—The patient was supposed to be dying by friends; medi-
cines had been discontinued since 12 o’clock last night.
I now explained to the friends that it was possible that a copious injection
might overcome the obstruction, and afford relief ; this, I said, will occasion
much pain, but we certainly ought not to let the patient die without making
at least one more effort to afford relief. Of the many present, I selected two
women of nerve and decision, to carry out my directions. With a pump, I
ordered them to inject tepid water into the bowels, so long as they could pre-
vail upon the patient to endure it This they did, and returned to me soon,
informing me that they had injected only about a pint. The patient’s suf-
ferings, they said, were extreme. Less had been accomplished than I had
expected.
I now took the pump myself, closed the door against spectators, and com-
menced injecting, entreating the patient to endure to the utmost, as this was
her only hope, This she did, for a time, but soon her shrieks and groans
became heart-rending. Her husband and son now rushed into the room,
and commanded me to desist from further attempts to relieve the patient;
which 1 did only after at least two quarts had been injected. The friends
evidently looked upon me as a personification of brutality. Though con-
scious of having done my duty, I felt confident I had lost the confidence of
the many present, and that I had better have let the patient died, at least
without the last effort for relief.
About an hour after this, to my great joy, the bowels moved freely and
repeatedly, to the no inconsiderable relief of the patient. From this time on,
the only laxative required was a little yeast. The patient, however, was not
destined to recover. She died about two weeks later, seemingly from an
inability to rally from the extreme prostration. Peritoneal inflammation
was, doubtless, the cause of death, which, in a person of less years, and
under more favorable circumstances, might have been cured.
I can not avoid the conviction, that if the copious injections, with a
view of forcing the obstruction, had been resorted to earlier, the result might
have been different.
Frewsburg, Chatauque Co., N. Y.
Anaesthetics.—We quote the following article from the Lancet for Octo-
ber 16, 1858, and we have no doubt that all it asserts is true. Indeed, we
have looked over the late Dr. Snow’s work with much interest, and can
heartily join in the recommendation of the Lancet, to give this “ valuable
monograph” a “thoughtful perusal.” Notwithstanding all that is said,
however, even by the highest authority, we believe that the time is not far
distant when chloroform will be abandoned as an anaesthetic agent. We
again ask, why use an article of such uncontrollable power, when we have
an equally reliable one in ether, and one which is entirely safe ? It is no-
torious, that under the best conditions, and in the most careful hands, chloro-
form is a dangerous weapon. It is only lately that Mr. Erichsen, high sur-
gical authority, has stated both pubilcly and privately, that he uses chloro-
form as he wonld take an express train, ignoring, or risking, the dangers
of extra velocity. Now, is it not better to travel on the anaesthetic track a
little more slowly, and be safe in our journey ? Mr. Erichsen himself inti-
mates as much as this; and the evidence of every day only confirms us in
the opinions we have expressed.
“ The frequent recurrences of death from the administration of chloroform
during the last few weeks has induced a feeling of uneasiness and distrust
of this agent both in the profession and out of it. It is very certain, how-
ever, that a great part of this feeling might very advantageously be trans-
ferred to the mode of administration which is still in Vogue. It must not
be forgotten that, in all these cases, the chloroform was administered loosely
on a handkerchief, and in more than one instance, as has been proved, it was
of a thickly-woven texture, which did not allow of a free passage of air.
This mode of administration is, we think, most unjustifiable. The experi-
ments of the late Dr. Snow conclusively showed the necessity of carefully
regulating the proportion of vapor in the air inspired. We strongly recom-
mend to thoughtful perusal the valuable monograph on the subject of
Anaesthesia which he has bequeathed to the profession. No one can rise
from reading this valuable digest of a wide experience and the observation
of ten years of scientific and practical labor, without a feeling of regret that
so much carelessness should still prevail in the administration of this most
potent vapor, and a sense of the necessity for a more extended instruction in
the principles of anaesthetization. We commend the following sentences to
the very careful consideration of surgeons:—
“ The great point to be observed in causing insensibility by any narcotic
vapor, is to present to the patient such a mixture of vapor and air as will
produce its etfeets gradually, and enable the medical man to stop at the
right moment. Insensibility is not caused so much by giving a dose as by
performing a process. Nature supplies but one mixture of diluted oxygen,
from which each creature draws as much as it requires; and so, in causing
narcotism by inhalation, if a proper mixture of air and vapor is supplied,
each patient will gradually inhale the requisite quantity of the latter to cause
insensibility, according to his size and strength. It is, indeed, desirable to
vary the proportions of vapor and air, but rather according to the purpose
one has in view, whether medicinal, obstetric or surgical, than on account of
the age or strength of the patient; for the respiratory process bears such a
relation to the latter circumstances, as to cause each person to draw his own
proper dose from a similar atmosphere in a suitable time.’
“ The proportion of chloroform most suitable to produce insensibility, Dr.
Snow found to be about four cubic inches of vapor, or rather more than five
grains of chloroform to one hundred cubic inches of air. With a properly-
arranged inhaler, it is easily possible to supply the vapor in this fixed position.
This simple precaution would rob chloroform of nearly all its terrors and its
dangers. L is a very surprising consideration, that while the niceties of
surgical manipulation are invaribly attended to with the utmost care, the
production of anaesthesia is very frequently left in the hands of quite inex-
perienced persons. It is sad to see the issues of life and death treated with
indifference; it would be yet more so, if we were not well aware that the
fault is one of thoughtlessness, and that those who have fallen into it will
probably shrink from a similar error now that it has been pointed out so
forcibly by recent events.”—Boston Med. and Surg. Jouraal.
Warning to Invalids Visiting the Island of Cuba.—[We beg leave to
present our readers the following extract from a letter received from an
esteemed correspondent in Havana. It speaks for itself, and all medical men
who send patients to Cuba for relief, should be careful to make every effort
to secure them from the imposition pointed out.—-Eds.]
If you like to take the trouble to give notice to your readers, that such
of their patients and other invalids as come to Havana shall be counseled in
respect to localities best suited to their conditions, or if so ill as to require
private lodgings, or residences separate from the crowd and discomfort of
hotels, it will give me much satisfaction to be of service to them in any way
that may be in my power, and to carry out every judicious plan of treat-
ment that may be recommended by their physicians.
I do not wish to be understood as advertising myself, or as soliciting cases,
but the custom with infirmaries and several physicians, of employing run-
ners and of paying commissions to catch strangers, renders it suitable that
the profeasion be advised of the indecent usages and its objects; and the
more so, that the parties guaranty quick and radical cures of diseases in the
most desperate circumstances, stipulating beforehand the amount of money
to be paid, usually from one to five thousand dollars, and the largest part in
advance that can possibly be obtained. The patient is always required,
under the pretext of air, diet, or something else, to take lodgings in some
family in which the physician confides, and the members of which, and also
their friends, are instructed in the part they are to act in the conspiracy.
At the proper time, an interpreter is at hand; at other times, the patient is
surrounded by persons he cannot understand or make understand him: the
thieves soon seize his pile, and proceed in the process of fleecing their vic-
tim, whose effects in the end fall short of paying his bills; the flattering
arrangement has cost the unfortunate patient his last dollar, with no result
but a heartlsss robbery, and chagrin and bitter disappointment. Instances
of this kind of financiering occur every season, and it happens, also, if the
funds of the victim do not hold out to pay every dollar, and the regular
course of the disease, or some other cause, has ended in the death of the
patient, his acquaintances and countrymen must provide for his interment,
or see his lemains conveyed in the common dead-cart to the cemetery, and
deposited without coffin or any covering in the ditch or common receptacle
of the dead from hospitals, prisons, and places of execution. Strangers
who are invalids, should consult their Consul, or some countryman of estab-
lished character, in respect to whatever they propose to do, particularly in
respect to medical attendants, and all matters involving important interests,
with which they themselves are perfectly familiar. There is no need of any
person falling into the hands of medical thieves and robbers. In Havana,
there are great numbers of professional gentlemen, whose spotless honor
and high attainments would lose nothing by any test or comparison, and
whom money could not induce to guarantee the cure of a disease already
advanced to the point which makes it necessarily mortal; but these do not
employ runners or whippers-in, or divide their fees in commissions.
The credulity of invalids is notorious. Men of the best capacity, the
highest attainments and stations, heads of the law and church, allow them-
selves to fall victims to the most grovelling impostures, and dupes of the
vilest quackery. The best understandings are betrayed by credulity and
fear, or importuned out of their senses by designing rogues, officious spectators,
as well as by honest-meaning friends. If we consider how intensely life
and health engage the hopes and fears of all, the blindest judgment in her
vain groping for something to ward off despair, and her idiotic trust in igno-
rance and knavery ought rather to be matter of regret and commiseration,
than of surprise and derision; and allowance should be made for the effects
of sickness, for impatience from sufferings unrelieved, perhaps unrelievable
by any skill.—New Orleans Medical News and Hospital Gazette.
				

